# Silicone Implant Versus Silicone Implant Assisted by Stromal Enriched Lipograft Breast Augmentation: A Prospective Comparative Study

**DOI:** 10.3390/medicines7050028

**Published:** 2020-05-19

**Authors:** Aris Sterodimas

**Affiliations:** Plastic & Reconstructive Surgery Department, Metropolitan General Hospital, 264 Mesogeion Avenue, 15562 Athens, Greece; aris@sterodimas.com

**Keywords:** stromal enriched lipograft, breast augmentation, composite augmentation, fat grafting, breast implant, mastopexy

## Abstract

**Background:** Implant-assisted breast augmentation is among the most performed surgeries performed by plastic surgeons today. This prospective study evaluated the patient satisfaction and complication rates using high-profile round silicone implant alone (traditional breast augmentation) Group A versus the high-profile round silicone implant assisted with stromal enriched lipograft (composite breast augmentation) Group B. **Methods:** A total of 50 female patient candidates to undergo breast augmentation between January to September 2017 were randomly assigned to either group. The periareolar technique for breast augmentation and the subfascial plane were used in both groups for the insertion of the high-profile round silicone implants. The stromal enriched lipograft (SEL) was used in Group B for the preparation of the autologous fat grafting to the breast. The satisfaction of each patient with body appearance following breast augmentation was rated using an already published scale of 1–5. The rate of complications was analysed. **Results:** AS performed all the surgeries. In Group A, the age range was between 19 and 48 years (mean of 22.5 years). In Group B, the age range was between 20 and 47 years (mean of 24.1 years). The average BMI of Group A was 24 m/kg^2^ and 23 m/kg^2^ of Group B. Patient satisfaction meta-analysis for Group A and B at 12 months shows that patients in Group B expressed a satisfaction that is superior when compared to Group A patients. The ability to camouflage the implant could explain the higher satisfaction rates in Group B. The rate of complications appears similar in both groups. **Conclusions:** Composite breast augmentation using a combination of round high-profile implants and SEL in breast augmentation can achieve a higher patient satisfaction and aesthetic outcome as compared to the round high-profile breast augmentation alone. The technique is safe, simple and fast with low complication rates. Large multicentre, controlled, prospective studies need to be performed to further confirm the favourable results that were observed in this study.

## 1. Introduction

The breasts are one of the most important characteristics of femininity [1]. Breast augmentation surgery with implants is one of the most common aesthetic surgical procedures performed worldwide. In patients that will undergo breast augmentation, various factors need to be addressed like the age of the patient, the breast volume, the tissue coverage, the type and the size of the implant, and the resulting scars. Breast augmentation is among the procedures that are most performed worldwide [2]. There are reports, though, that revision surgery due to non-satisfactory results or complications like implant show, rotation, capsule contracture and implant malposition need to be addressed [3]. Many patients also notice the unnatural result of the breast augmentation achieved when only silicone implants are used.

Recently, there are several published articles that demonstrate that stromal enriched fat grafts provide a good cell viability proving that it is an effective and safe and procedure in just one treatment session with high patient satisfaction [4,5,6,7,8].

This is a prospective study to analyse the rate of complications and the rate of satisfaction following breast augmentation using the technique of inclusion of silicone implant alone (traditional breast augmentation), Group A, versus the technique of inclusion of the implant assisted with SEL (composite breast augmentation), Group B.

## 2. Patients and Methods

Fifty female patients, candidates for breast augmentation, were included between January to September 2017 in this study. Breast hypoplasia was the inclusion criterion for random assignment into either to Group A or Group B, and they all underwent silicone implant breast augmentation. Patients in Group A had the traditional breast augmentation using textured round silicone implants. Patients in Group B underwent the composite breast augmentation using textured round implants combined with SEL. The amount of fat to be grafted in Group B patients was chosen based on the patient’s preference for the size of the breast. The surgeon and each patient discussed the different silicone implant sizes and the amount of fat to be injected on each site of the breast in order to camouflage the implant and give a more natural final result. After the silicone implant was inserted, the areas of the breast that needed to be fat grafted were marked, and SEL was injected. All patients signed an informed consent form to participate in the study, which was approved by the Ethics and Scientific Committee of IASO General hospital EC 29/8-9-2016. Date of approval: 8 September 2016. The criteria for inclusion in this study were female patients that were ≥18 years old who had not been submitted previously to any breast surgery and requested augmentation for cosmetic purposes. The exclusion criteria were female patients with a previous history of breast cancer, mammoplasty, breast augmentation with or without the use of silicone implant, serious systemic diseases and patients that did not agree to be randomly assigned to the study. The demographics of the patients were analysed based on the age, body mass index (BMI), smoking and the past medical history. The body appearance satisfaction following breast augmentation was rated on a scale of 1–5 where 1 was poor, 2 was fair, 3 was good, 4 was very good and 5 was excellent. This specific scale has already been published in peer reviewed articles [9,10]. The evaluation was done 1 year after the surgery. The rates of complications are shown for each group.

IBM SPSS for Macintosh version 21.0 (SPSS, IBM Corp., Armonk, NY, USA) was used for statistical analysis. An a priori power analysis was used in order to have 80% power and an α level of 0.05, capable of achieving a significant PSR difference. It was calculated that 50 patients were needed.

The patients’ satisfaction rates (PSR) at 12 months were summarized as means (SDs) for each group. To compare differences in PSRs between the 2 groups, mixed-effects regression models were applied, with dependent variables being the PSR values and the predictors that correlate with these PSR values at 12 months postoperatively, using Rubin’s rules. Adjusted means of PSR values were performed using SAS platform, version 9.4, and *p* < 0.05 was set as the statistical significance.

### Surgical Technique

**Figure 1:**
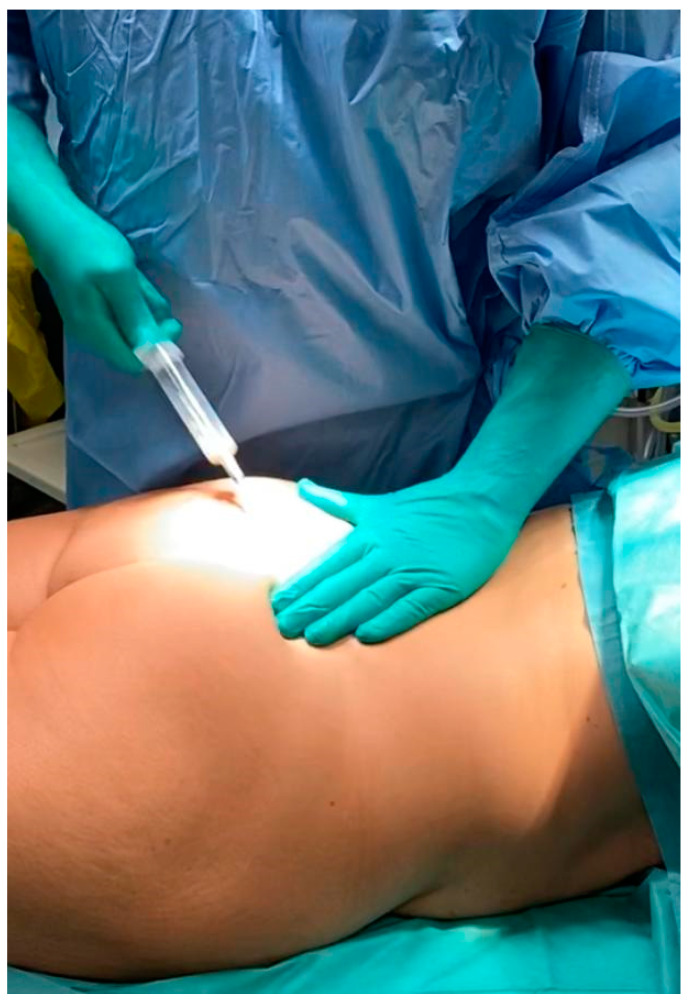
Liposuction performed by syringe method.

**Figure 2:**
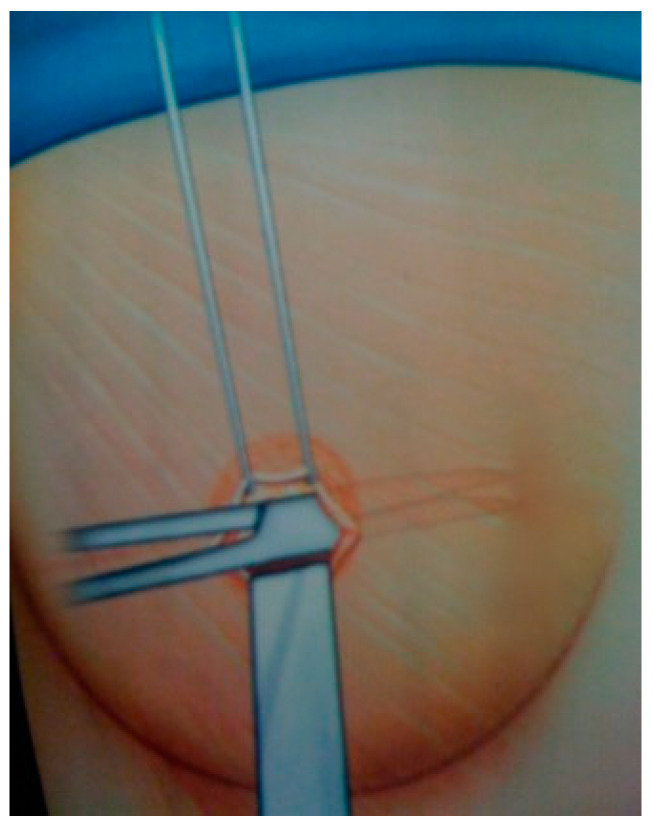
Through inferior periareolar and breast gland dissection is done for both groups.

**Figure 3:**
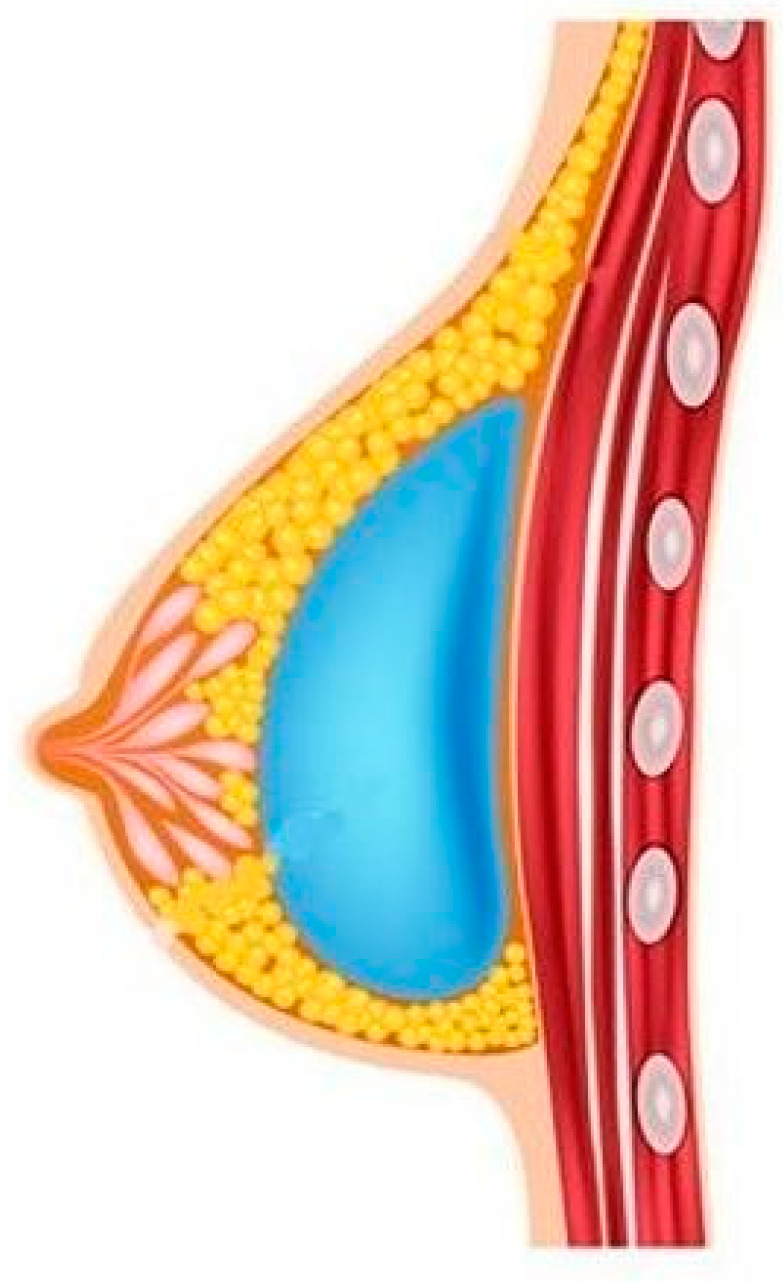
Subfascial plane creation is done.

**Figure 4:**
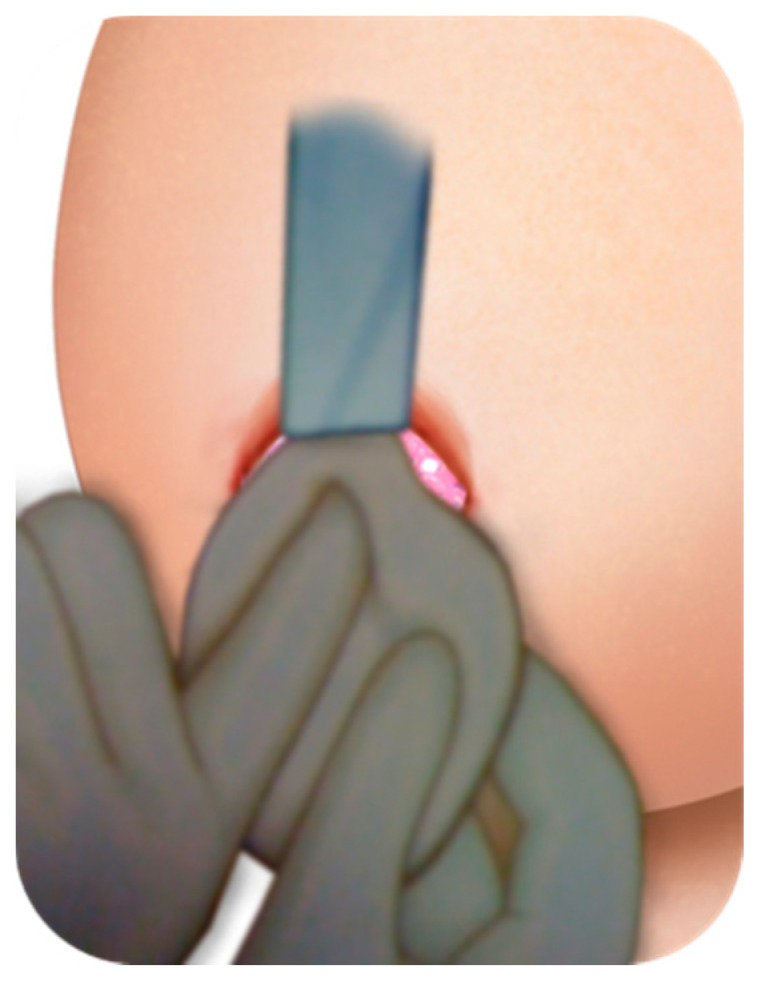
Inclusion of the silicone implant.

**Figure 5:**
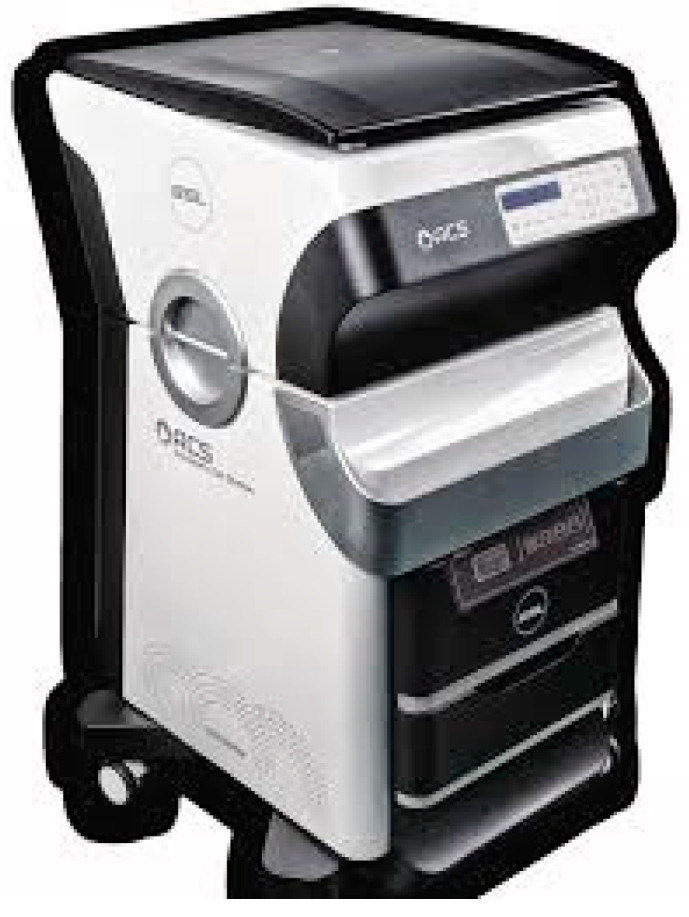
Fat is treated using the Automatic Cell Station (ACS), produced by BSL Ltd., Seoul, Korea.

**Figure 6:**
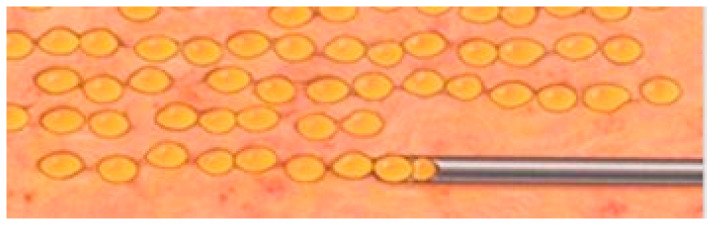
Drop by drop technique of laying the fat in the subcutaneous breast plane using retrograde injection.

**Figure 7:**
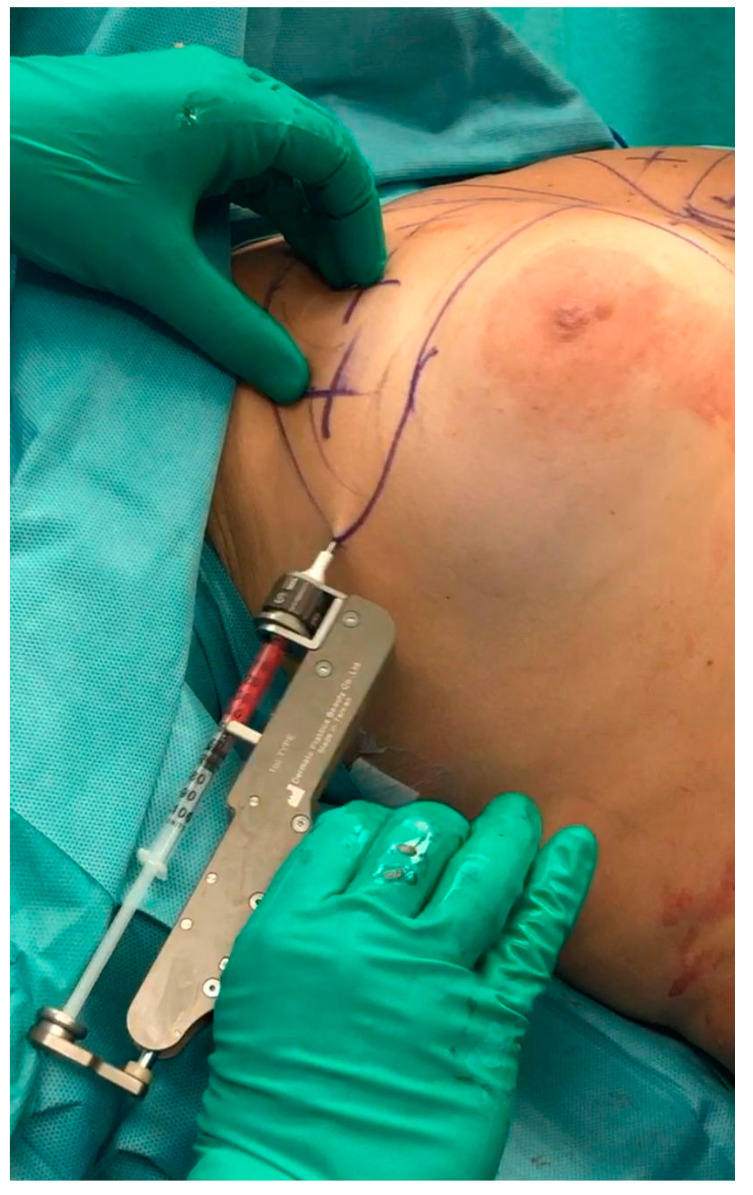
The Maft Gun, Dermato Plastica Beauty Co, Ltd., Taipei, Taiwan is used in order to perform the injection.

1. The specific body areas chosen together with the patient in order to do liposuction are marked while the patient is in standing position for Group B.
2. Sedation and epidural anaesthesia are done for both groups. Areola is marked using the infra-areolar approach. Local infiltration with adrenaline (1:500,000) is then done. For Group B patients, a 4 mm blunt cannula attached to a 60-cc syringe is used for liposuction (Figure 1).
3. Incision using the Webster marking, inferior periareolar, (Figure 2) and using a perpendicular incision through the breast gland is done for both groups. The gland is divided, and thorough haemostasis is performed.
4. The subfascial plane is dissected, and a pocket for silicone implant insertion is created (Figure 3).
5. Inclusion of the implant is done (Figure 4).
6. Closure using 3-0 Monocryl (Ethicon, Sumerville, NJ, USA) interrupted sutures for the glandular and subdermal planes is done. Continuous Monocryl 4-0 suture is used in the intradermal level. No drains were used.
7. Fat aspiration is treated using the Automatic Cell Station (ACS), produced by BSL Ltd., Seoul, Korea (Figure 5). The SVF is derived from 2/3 of liposuctioned fat. Collagenase type II (Sigma, St. Louis, MO, USA) is used for processing the fat for 45 min at 37 °C. The SVF is dissociated by centrifugation at a speed of 1200× *g* for 5 min according to the protocol already published [11]. The remaining 1/3 of fat is washed with 0.9 % NaCl until the fat is concentrated and purified. The SVF rich in ADSCs is mixed with the concentrated fat and transferred into 10 mL syringes ready for injection.
8. The breast consists of 4 quadrants, and a 1.9 mm cannula connected to a 10 mL syringe is used to inject in different planes of the subcutaneous breast tissue with multiple passes in a fan shaped mode. A drop by drop technique is used as the cannula is withdrawn, in order to avoid complications and to obtain a significant clinical outcome (Figure 6). The Maft Gun, Dermato Plastica Beauty Co, Ltd., Taipei, Taiwan, is used in order to perform the injection (Figure 7). The entire breast in Group B patients is injected according the preoperative design.
9. Steri-strips (3M’s Nexcare, USA) are applied to the incisions, and a special bra needs to be worn for 1 month postoperatively. The patients in both Group A and B were prescribed paracetamol 500 mg qd and Nimesulide 100mg bd for the first postoperative days. Prophylactic antibiotic Zinadol 500 mg capsules are prescribed for a week postoperatively. Both Group A and B patients underwent the surgical procedures as day patients. No patient needed to stay overnight, and the postoperative visits for both groups were the same.

## 3. Results

AS performed the surgical procedures in both groups. The age ranged from 19 to 48 years (mean of 22.5 years) in Group A. The age ranged from 20 to 47 years (mean of 24.1 years) in Group B. The average BMI for Group A was reported to be 24 m/kg^2^ and 23 m/kg^2^ for Group B. Ten patients were smokers in Group A and twelve in Group B. The mean volume of implant inserted was 285 cc (range, 195–385 cc) in Group A and 235 cc (range, 195–315 cc) in Group B. The mean volume of SEL inserted in Group B was 75 cc per breast (range, 35–110 cc). The average time for a traditional breast augmentation in Group A ranged between 60–110 min. The average time for the composite breast augmentation in Group B ranged between 75–120 min. In Group A, 4 patients had scar hypertrophy, and one patient breast asymmetry. No infection was reported and no hematoma or capsular contracture. Scar hypertrophy in 2 patients was reported in Group B. No capsular contracture, hematoma or infection were noted in group B. No implant dislocation was observed in this series of patients. All patients in both groups had only one operation, and no secondary intervention was needed. No oil cysts were noted in any patient including smokers and non-smokers in Group B.

**Figure 8:**
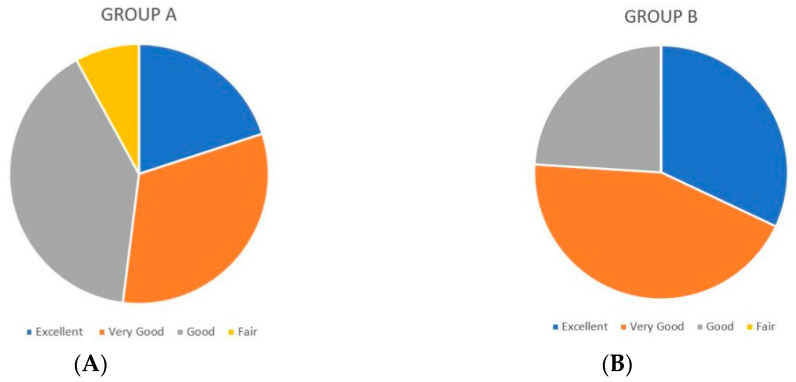
(**A**) Patient satisfaction rate after breast augmentation in Group A. (**B**) Patient satisfaction after breast augmentation and SEL in Group B.

At 12 months post operation, in Group A, 13 patients answered that their result was “very good” (8) to “excellent” (5); ten reported that their result was “good” and two “fair” (Figure 8A). At 12 months, in Group B, 19 patients answered that their appearance was “very good” (11) to “excellent” (8) and six that their appearance was “good” (Figure 8B). The average follow-up time for both groups was 2.3 years. The mean score for the patient satisfaction rate for Group A was 3.72 (SD = 2.29) out of 5 and 4.08 (SD = 2.29) for Group B. The parameters, in descending order, that were most important in this study were the breast symmetry, the final breast shape, and the resulting scar (*p* < 0.05).

### 3.1. Case 1

**Figure 9:**
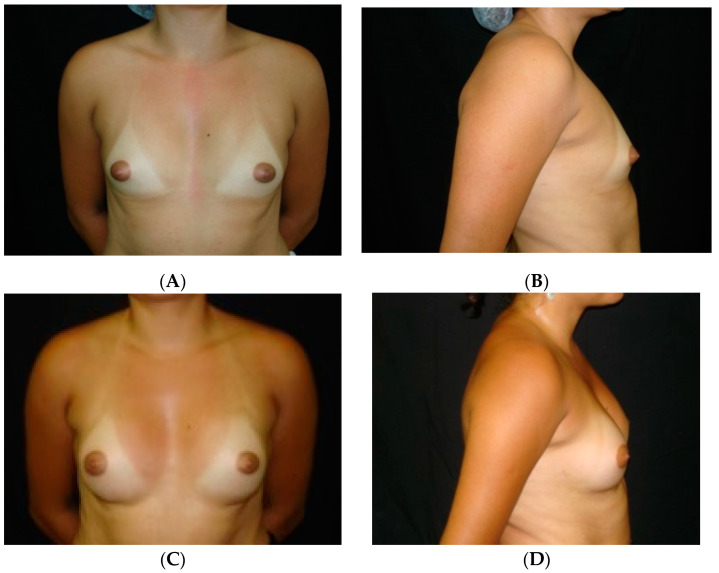
(**A**,**B**) A 24-year-old woman asking for breast contour enhancement. (**C**,**D**) Bilateral breast augmentation using round high-profile breast implants (195 mL). Photos were taken 12 months after the procedure, and her satisfaction at 12 months was reported as very good.

This 24-year-old female patient asked for breast contour enhancement (Figure 9A,B). She was randomly placed into Group A. Bilateral breast augmentation using round high-profile breast implants was done (195 mL). Postoperative views 12 months after the procedure are shown (Figure 9C,D). At 12 months she reported her aesthetic result as very good.

### 3.2. Case 2

**Figure 10:**
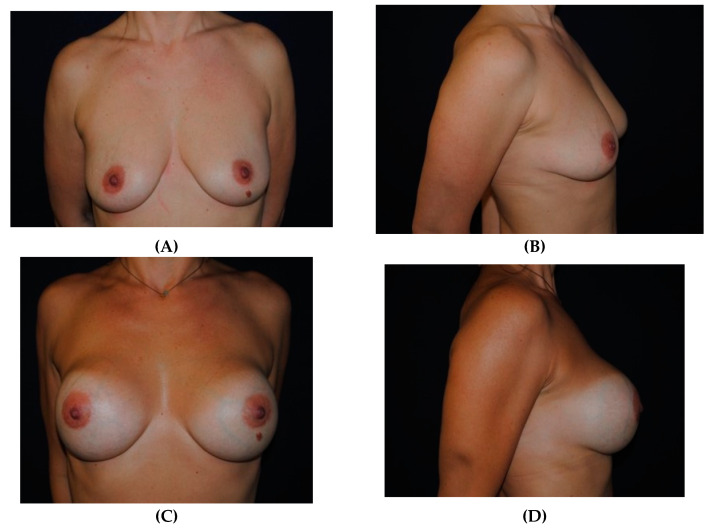
(**A**,**B**) A 38-year-old woman for enhancement of her breast contour. (**C**,**D**) Photos taken 12 months after bilateral breast augmentation using round high-profile breast implants (285 mL) with her satisfaction rated as excellent.

A 38-year-old female patient asking for breast augmentation (Figure 10A,B). She was randomly placed into Group A. She had round high-profile silicone implants placed bilaterally (285 mL). The postoperative result 12 months after the procedure is shown (Figure 10C,D). At 12 months, her result was reported as excellent.

### 3.3. Case 3

**Figure 11:**
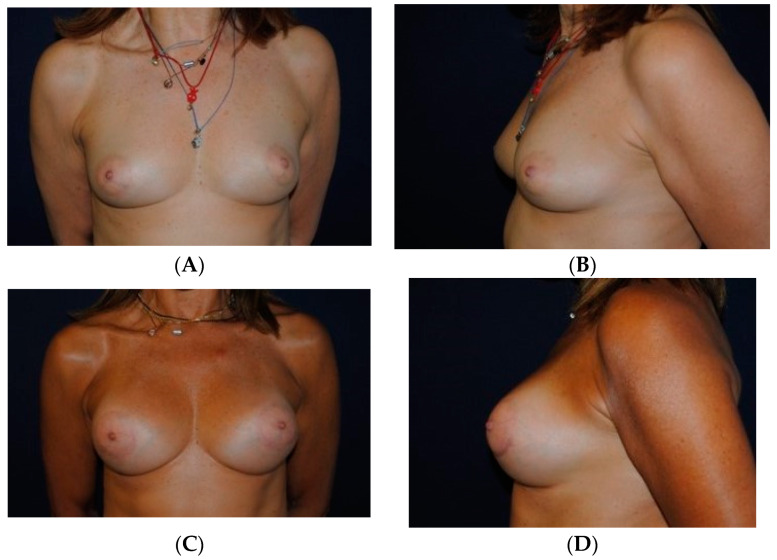
(**A**,**B**) A 47-year-old woman of Group B candidate for breast augmentation. (**C**,**D**) Postoperative photos of the 47-year-old after bilateral breast augmentation with round high-profile breast implants (285 mL) and SEL of 96 mL on the right breast and 106 mL on the left breast. Her satisfaction at 12 months rated as excellent.

A 47-year-old woman presented to our department for breast augmentation (Figure 11A,B). She was assigned to Group B. Bilateral breast augmentation was done with round high-profile breast implants (285 mL) and SEL of 96 mL on the right breast and 106 mL on the left breast. Twelve months after the procedure, the result is shown (Figure 11C,D). The satisfaction rate at 12 months was rated excellent.

### 3.4. Case 4

**Figure 12:**
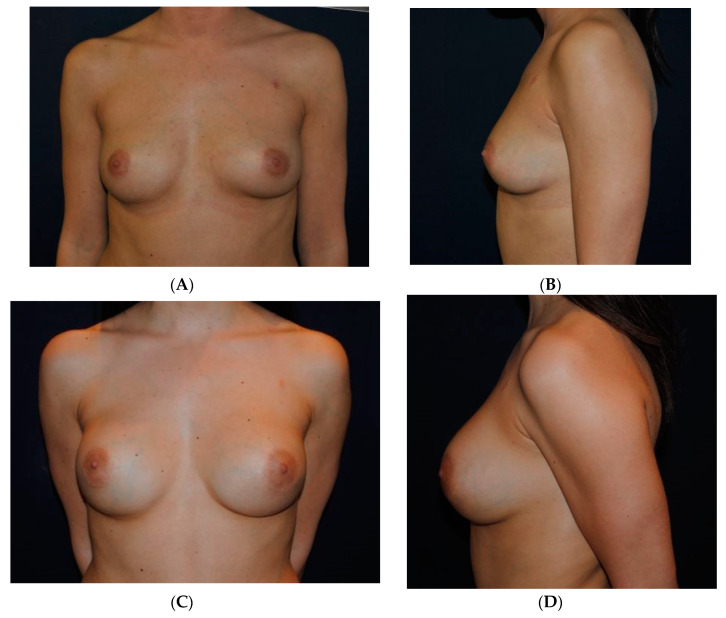
(**A**,**B**) A 25-year-old woman asking for a breast enhancement. (**C**,**D**) Photos taken postoperatively after bilateral breast augmentation with round high-profile breast implants (235 mL) and SEL of 56 mL on the right breast and 65 mL on the left breast. The patient is shown 12 months after procedure, and her satisfaction was rated very good.

A 25-year-old woman asked for a breast augmentation (Figure 12A,B). She was randomly assigned to Group B. Bilateral breast augmentation was performed by round high-profile silicone implants (235 mL) and SEL of 56 mL on the right breast and 65 mL on the left breast. Photos were taken postoperatively 12 months after the procedure (Figure 12C,D). At 12 months, her satisfaction was rated very good.

## 4. Discussion

Breast augmentation for aesthetic purposes has been reported to be important for patients’ self- attractiveness. An increase in quality of life has been also reported when patients have been submitted to breast augmentation as they report an increase in sexual satisfaction due to improvement physical appearance [12,13]. Different factors and criteria are to be considered in order for the final aesthetic result after breast augmentation to be successful [14]. The technique of subfascial breast augmentation is a technique that is considered safe and effective, delivering predictable results with a high patient satisfaction rate. The subfascial approach, when properly indicated, has the advantages of subglandular and submuscular implant position together without the drawbacks associated with each [15,16,17,18]. 

Patients from both groups expressed their satisfaction with the breast augmentation procedures in this study. The cost of the composite breast augmentation (Group B) was 35% higher as compared to the traditional breast augmentation (Group A), as Group B patients underwent liposuction and their adipose tissue had to be processed using the SEL protocol. This cost difference is due to extra surgical suite use and due the use of consumables for the SEL protocol. A statistical difference on the patient satisfaction rate was noted between Groups A and B. Meta-analysis of the patient satisfaction at 12 months shows Group B rate to be superior when compared to Group A. This could be explained due to the more natural appearance of the breast in Group B, as the implant offers breast projection and the SEL offers camouflage of the implant. A smoothened upper pole and lower pole in Group B was noted. No difference in the complication rate between the two Groups was noted. The implant was placed in the subfascial pocket in order to be covered by the fascia. SEL did not increase the infection rate in Group B as compared with Group A complication rates. Breast augmentation assisted by SEL was feasible with high satisfaction and low complication rates.

The subcutaneous tissue of the breast has a rich blood supply, and fat grafting should be performed in this plane, increasing the chance of fat cells survival and providing a better graft take [15]. The retrograde drop by drop injection technique used by AS in different tunnels guarantees a uniform fat placement and at the same time prevents the creation of encapsulated masses due to bolus fat injection which could become calcified and may interfere with future breast screening [19]. There are patients who request a big volume breast, and in those cases, the combination of breast implant insertion with stromal enriched lipograft is advisable. The intermammary distance and the medial symmetry between the breasts are important parts of the outcome of surgery and have a strong bearing on patient satisfaction. The division of the breast into four cosmetic units needs to be done and fat grafting of these breast subunits should be performed. This guarantees proper execution without overfilling of the breast subunits. The SEL was inserted a in the medial and the lateral sides of the breast in order to camouflage the edge of the implant and to produce a more natural final result.

After performing fat grafting to the breast, it has been reported that benign calcifications, oil cysts and parenchymal densities as well as cystic lesions can be expected, and it is the duty of an experienced radiologist to differentiate them and suggest further imaging if needed [20]. After breast reduction procedures, fat necrosis resulting in cyst formation can be depicted in annual mammograms [21]. The major complications reported after fat grafting to the breast are due to technical errors and improper preparation of the fat to be injected [22]. One of the most common complications of breast fat injection are necrotic cysts which appear benign in ultrasound, in mammography and in magnetic resonance imaging (MRI) diagnostic tests [23]. Nowadays, radiologists can use diagnostic modalities that can permit to distinguish between benign necrosis of fat tissue and cancer. The evaluation of breast lesions after fat grafting to the breast requires experience of the radiologists in mammogram and ultrasound examinations. MRI may be needed in selected cases. The appearance of breast fat that has been grafted has a lower T1 signal in comparison with the normal breast fat, and T2 signal of fat graft appears higher than breast subcutaneous fat. This is due to the breast fibrosis that can appear after fat grafting to the breast [24]. A recent study has documented that calcifications due to fat grafting to the breast and those caused by malignant lesions can be easily distinguished [25]. Postoperative follow-up of breast imaging is not affected by fat grafting, and in case there is any lesion that needs to be biopsied, an FNA can be performed [26]. Normal recommendations for breast screening can be followed in the patients that underwent fat grafting as in the normal population [27]. Studies that include more than 2000 patients that underwent fat transplantation in the breast have not reported increase in cancer [28]. Breast ultrasound is used for follow-up of the fat grafting procedure in the breast and has been reported to be very accurate in detecting lesions [29]. Patients with a susceptible lesion that is discovered in less than a year after fat grafting to the breast need further evaluation.

There have been case reports in the literature that raise concerns about injecting adipose-derived stem-cell-enriched lipograft to the breast and the possibility of triggering precancerous tissue activation. The available published studies have proven, though, that fat grafting to the breast is safe for aesthetic and reconstructive purposes [28,30,31]. SEL to the breast is a procedure that needs to be done by surgeons who have extensive experience in this field. All the patients who took part in this study were informed that there may be an increased risk of calcification and cyst formation, and all patients were examined in a follow-up by mammography and ultrasound according to the guidelines established worldwide.

Patients from both groups expressed their satisfaction with the breast augmentation procedures in this study. The cost of the composite breast augmentation (Group B) was 35 % higher as compared to the traditional breast augmentation (Group A), as Group B patients underwent liposuction and their adipose tissue had to be processed using the SEL protocol. This cost difference is due to extra surgical suite use and due the use of consumables for the SEL protocol. A statistical difference on the patient satisfaction rate was noted between Groups A and B. Meta-analysis of the patient satisfaction at 12 months shows Group B’s rate to be superior when compared to Group A. This could be explained due to the more natural appearance of the breast in Group B, as the implant offers breast projection and the SEL offers camouflage of the implant. A smoothened upper pole and lower pole in Group B was noted. No difference in the complication rate between the two groups was noted. Breast augmentation assisted by SEL was feasible with high satisfaction and low complication rates.

Most of the studies published today have reported on autologous fat grafting techniques in patients who have undergone breast reconstruction following a mastectomy or breast conserving surgery. This study has been designed prospectively, with data collected pre-operatively and post-operatively. There are very limited studies focused on composite breast augmentation for cosmetic purposes. To our knowledge, this is the first study that compares the traditional breast augmentation using silicone implants to the composite breast augmentation using silicone implant and stromal enriched lipograft. This is also the first study that proves that the composite breast augmentation bears similar complication rates when compared to the traditional breast augmentation. Additionally, there is a significantly higher patient body satisfaction rate in the series of composite breast augmentation when compared to the traditional breast augmentation. This study and its results are of clinical importance for the female patients that would like to undergo breast augmentation in order to help them to make an informed decision. This prospective study has limitations. The total number of the patients was only 50, and they were evaluated using the body appearance satisfaction scale expressed as a subjective patient rate. The average follow-up time was 2.3 years, which is a short follow-up time. Large multicentre, controlled studies need to be done in order to confirm the favourable results seen in this study. The Breast-Q questionnaire could have been used in this study, and this may be a drawback of this study. The composite breast augmentation can be suggested in patients who do not have enough tissue coverage in the breasts. The mismatch between soft tissue coverage and final breast size may result in palpability and edge visibility of the implant and most of the times rippling can be observed. Another indication for composite breast augmentation is the breast asymmetry, where the use of different sizes of implants may not fully correct the asymmetry, and the need of fat grafting is needed for a more precise result. 

## 5. Conclusions

In this study, the composite breast augmentation appeared to be superior when compared to the traditional breast augmentation in terms of the aesthetic outcome. A statistically higher patient satisfaction was noted in the composite breast augmentation group. The complication rate was the same in both groups.
